# Localized primary melanoma of male urethra with a 4-year follow up

**DOI:** 10.1016/j.eucr.2021.101702

**Published:** 2021-05-10

**Authors:** C.R.T. Burity, S.B. Linica, R.D. Saade, F.T. Ferreira, L. Schultz, E.S. Bezerra, H.C. Franco, R.A. Oliveira, M.V.S. Costa

**Affiliations:** a Dr. Mário Gatti Municipal Hospital, Campinas, São Paulo, Brazil; b Advanced General Surgery, Santa Bárbara Hospital - São Leopoldo Mandic Medical School, Santa Bárbara D'Oeste, São Paulo, Brazil; c Preceptor of the Medical Residency in Urology, Dr. Mário Gatti Municipal Hospital, Campinas, São Paulo, Brazil; d Chair Man of the Medical Residency in Urology - Dr. Mário Gatti Municipal Hospital. Campinas, São Paulo, Brazil; e Anatomophatology Institute (IAP), Santa Bárbara D'Oeste, São Paulo, Brazil; f Chair Man of the Medical Residency in Advanced General Surgery, Santa Bárbara Hospital - São Leopoldo Mandic Medical School, Santa Bárbara D'Oeste, São Paulo, Brazil; g São Leopoldo Mandic Medical School, Campinas, São Paulo, Brazil

**Keywords:** Melanoma, Urethra, Male

## Abstract

The objective is to perform a case report of primary melanoma of urethra in a male patient from a Teaching Hospital in Campinas, SP - Brazil. Male, 79 years of age, with blackened lesion on the meatus of the urethra with 1.5 cm of dimension, that could be palpated on the fossa navicularis region associated to an irregular lesion on the region of and the foreskin. The biopsy showed a malignant neoplasia. A partial penectomy with oncological margin of 2 cm was performed with a bilateral deep inguinal linfadenectomy. A follow up showed no recurrence of the disease.

## Introduction

Melanoma is an aggressive tumor with high rates of recurrence and progression. They are malignant tumors arising from melanocyte pigmented cells, that can be mostly cutaneous, but can also occur in various extracutaneous sites where pigment cells are present, such as mucosas, albeit rare.^1^ Malignant melanomas of the genitourinary tract account for a quarter of mucosal melanomas. The urethra is considered the most frequent site in the urinary tract, but it accounts for less than 1% of all melanoma and about 4% of urethral cancer.

The rarity of the disease often represents a limit to the participation of patients in clinical trials. In fact, studies with immune checkpoint inhibitors, approved in recent years for melanoma treatment, are still underway for mucosal melanoma and. So far, results, despite favorable, have been based on small retrospective series.^2^

## Case report

**Fig. 1:**
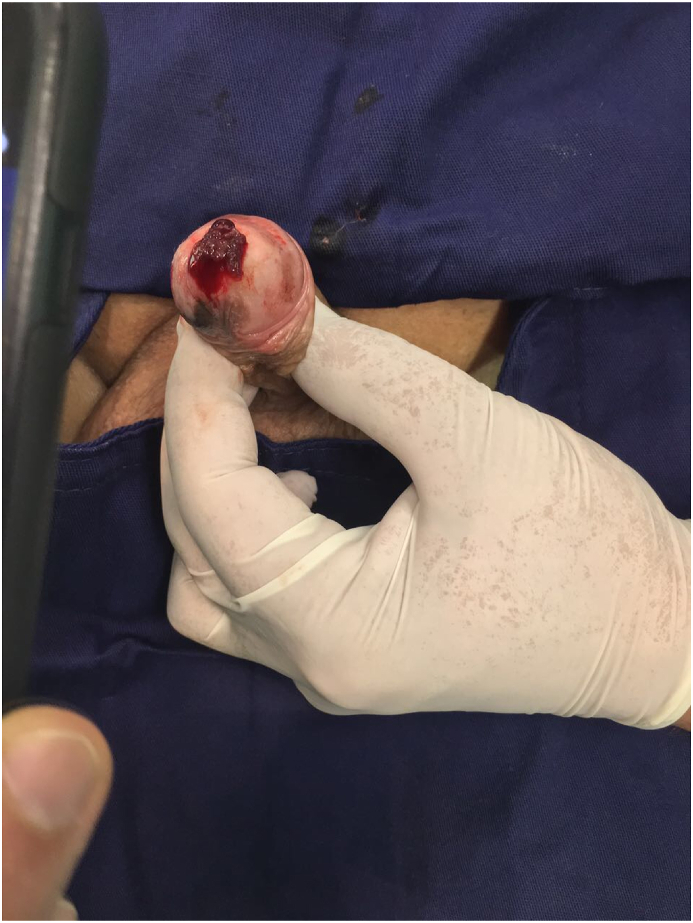
Physical examination: slightly penile compression exposes a vegetating mass insinuating from the male distal urethra (Tumoral mass localizated in the navicular fossa).

A 79 yo white male, married, with baseline hypertension, presented for urological consultation due to an altered urinary stream and a visible dark penile lesion on the glans. He noted it 6 moths prior and reported progressive growth. At physical examination, there was a vegetant blackened lesion on the meatus of the urethra, with 1.5 cm, that could be palpated on the fossa navicularis, associated with an irregular lesion on the foreskin (Fig. 1). The urinary stream was bifid and hemorrhagic. There were no palpable inguinal nodes.

An incisional biopsy was performed, revealing a proliferation of undifferentiated cells organized in solid or insular patter, with predominant spindle morphology. Nuclei were atypical, with conspicuous nucleoli, and frequent mitotic figures (some of them atypical), and an eosinophilic cytoplasm, sometimes with scant brown pigment. As findings were suspicious for melanoma, an immunohistochemical study was performed, which showed positive expression of melanocytic markers (S-100, HMB45 and Melan A).

**Fig. 2:**
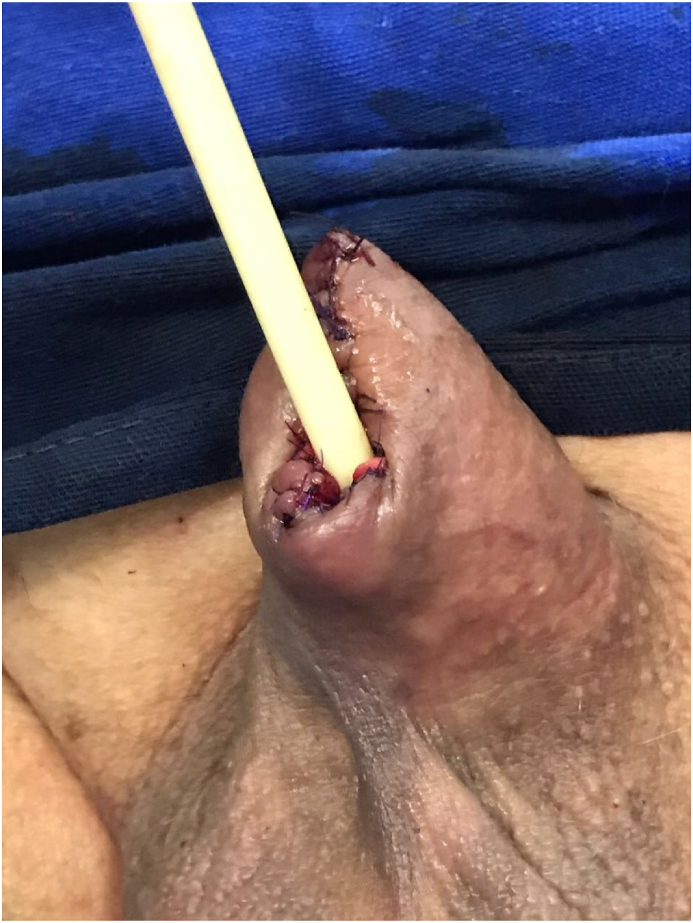
Postoperative aspect of partial penectomy and meatoplasty: Minimally invasive treatment of primary glander urethral melanoma.

Subsequent clinical staging included thoracic and abdominal tomography with no related findings (chronic hepatopathy, bilateral simple renal cysts and colonic diverticular disease). A penile MRI reported no evidence of corpus cavernosum or spongiosum invasion. A partial penectomy with bilateral deep inguinal lymphadenectomy (modified Catalona's dissection) was indicated, with 2.0 cm margin. The surgery proceeded without complications and the patient kept a residual penile shaft of 5.0 cm (Fig. 2).

**Fig. 3:**
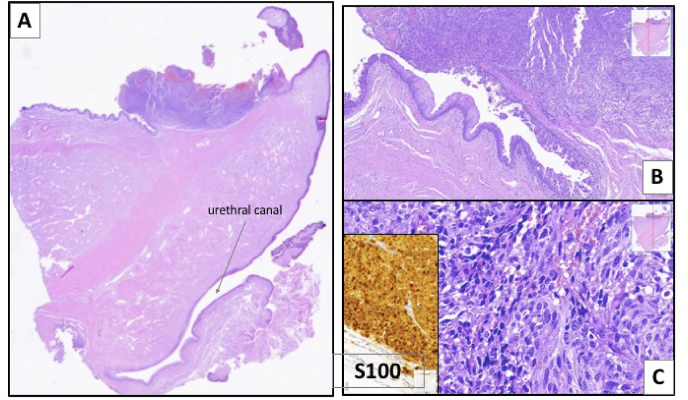
Histological images of the partial penectomy specimen: a hipercellular mass in the squamous-urothelial junction (A; H&E, 2x) with colonization of the adjacent mucosa (B; H&E, 10x). The mass is composed of highly malignant undifferentiated cells (C; H&E, 40x) which are positive for S100 (inset, immunohistochemistry), supporting the diagnosis of Melanoma.

Surgical pathology (Fig. 3) report described an ulcerated malignant melanoma involving the urethral mucosa (primary lesion) and extending to the glans, with irregular contours in a 1.4 cm maximum width, and 5.5 cm depth of invasion (Breslow) in the lamina propria, Clark level III. The main tumor was associated with an adjacent component of melanoma in situ that extended to the foreskin. Surgical margins were free of invasive or in situ component. There were no metastasis in 18 right and 15 left inguinal lymph nodes.

Post operatory evolution was satisfactory and patient has no signs of relapsed disease or progression at 24 months of follow up.

## Discussion

A large review of literature was performed (1962–2017), and a total of 54 studies reporting penile melanomas. The majority of the authors describe the urethral melanoma as an aggressive disease, with high rates of relapse and progression, with poor prognosis even at earlier stages. In our case report, there was an alteration on the urinary stream and eventual urinary retention, associated to urethral bleeding.^3^

The diagnosis was based on the histologic study complemented with immunohistochemistry, performed on an incisional biopsy of the lesion. Urethrocystoscopy may be necessary in cases where an urethral mass can be palpated. Treatment, however, is not a consensus. Some authors report radical penile surgery with extensive lymphadenectomy and even eventual cystoprostatectomy, while others have regarded partial penectomy with oncological margins and inguinal lymphadenectomy as sufficient intervention. More recent studies tend to suggest less aggressive procedures when possible, whereas radical surgeries are reserved for more advanced cases. Concerns about the preservation of anatomical structures and sexual function represent a limitation to the wide surgical margins.^4^

Melanomas of *uncommon sites* include cutaneous and mucosal lesions related to an unusual localization, in specific ethnic groups, in which exposure to UV radiation probably has no pathogenic role. The umbrella term “*sun-protected melanomas*” refers to mucosal, acral and genital melanomas, but it should be avoided, since these lesions are a heterogeneous pool regarding their genetic profiles and prognosis. The tumor mutational burden of mucosal melanomas is overall lower than that of cutaneous sun-exposed melanomas, which may impact in immunotherapy response rates. Also, a distinct pattern of chromosomal aberrations and a higher rate of copy number alterations have been reported in mucosal melanomas, as well as frequent KIT mutations in urogenital melanomas.^5^

Because of its infrequency, diagnosis and treatment of this illness are still a challenge. Primary melanoma of urethra is a rare and aggressive disease. Despite controversies about the extension of surgical intervention, early diagnosis and treatment are fundamental for a successful clinical evolution and patient survival.

## Source of founding

Not applicable.

## Declaration of competing interest

The authors declare that there are no conflicts of interest.
